# Lipid Peroxidation and Antioxidant Supplementation in Neurodegenerative Diseases: A Review of Human Studies

**DOI:** 10.3390/antiox9111128

**Published:** 2020-11-13

**Authors:** Snjezana Petrovic, Aleksandra Arsic, Danijela Ristic-Medic, Zorica Cvetkovic, Vesna Vucic

**Affiliations:** 1 Group for Nutritional Biochemistry and Dietology, Centre of Research Excellence in Nutrition and Metabolism, Institute for Medical Research, University of Belgrade, 11000 Belgrade, Serbia; snjezana570.imr12@gmail.com (S.P.); aleksandraarsicimi@gmail.com (A.A.); dristicmedic@gmail.com (D.R.-M.); 2 Department of Hematology, Clinical Hospital Center Zemun, 11000 Belgrade, Serbia; zokabora@yahoo.com; 3 Medical Faculty, University of Belgrade, 11000 Belgrade, Serbia

**Keywords:** neurodegenerative diseases, lipid peroxidation, antioxidant supplementation, Alzheimer’s disease, Parkinson’s disease, amyotrophic lateral sclerosis

## Abstract

Being characterized by progressive and severe damage in neuronal cells, neurodegenerative diseases (NDDs) are the major cause of disability and morbidity in the elderly, imposing a significant economic and social burden. As major components of the central nervous system, lipids play important roles in neural health and pathology. Disturbed lipid metabolism, particularly lipid peroxidation (LPO), is associated with the development of many NDDs, including Alzheimer’s disease (AD), Parkinson’s disease (PD), and amyotrophic lateral sclerosis (ALS), all of which show elevated levels of LPO products and LPO-modified proteins. Thus, the inhibition of neuronal oxidation might slow the progression and reduce the severity of NDD; natural antioxidants, such as polyphenols and antioxidant vitamins, seem to be the most promising agents. Here, we summarize current literature data that were derived from human studies on the effect of natural polyphenols and vitamins A, C, and E supplementation in patients with AD, PD, and ALS. Although these compounds may reduce the severity and slow the progression of NDD, research gaps remain in antioxidants supplementation in AD, PD, and ALS patients, which indicates that further human studies applying antioxidant supplementation in different forms of NDDs are urgently needed.

## 1. Introduction

Neurodegenerative diseases (NDDs) have become the major cause of disability and morbidity among older people worldwide due to the ageing society and the increased average life expectancy. Suffering from severe memory and behavioral impairment (dementia) and the loss of movement control (ataxia and paralysis), these patients need constant and long-term care, which is connected with huge economic and societal costs.

**Figure 1:**
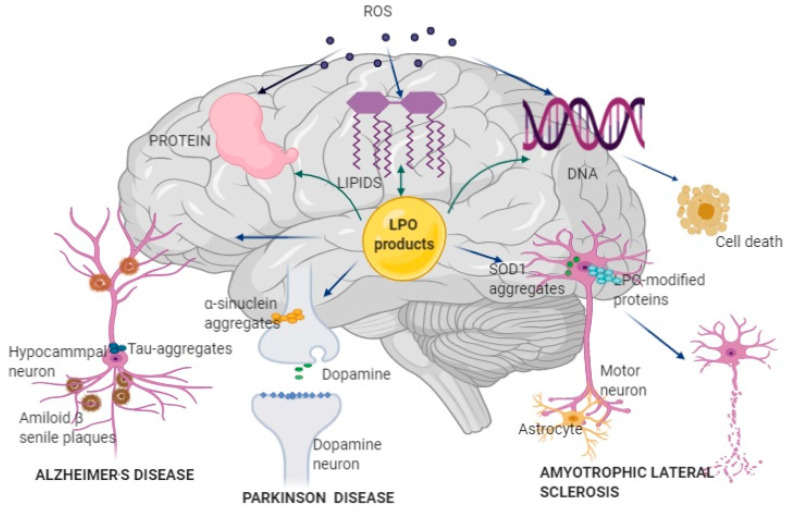
Pathophysiological mechanisms of Alzheimer’s disease (AD), Parkinson’s disease (PD), and amyotrophic lateral sclerosis (ALS) development and progression. Reactive oxygen species (ROS) produced by mitochondrial Cyt p-450, nicotinamide adenine dinucleotide phosphate (NADPH) oxidase, and lipoxygenase (LOX) attacks brain lipids, proteins, and DNA, further increasing oxidative stress. Oxidative damage of lipids results in the formation of lipid peroxidation (LPO) products, which further attack lipids, proteins, and DNA, impairing brain function. LPO can affect different types of neurons: in hippocampal neurons, LPO products bind to amyloid β peptide and form misfolded amyloid β peptide and amyloid β senile plaque, which disturbs nerve signaling and structure and induces AD; in dopaminergic neurons, LPO products induce generation and accumulation of misfolded α-synuclein, resulting in insufficient dopamine production and development of PD; in motor neurons, mutation of the superoxide dismutase 1 *(SOD1)* gene leads to the formation of misfolded SOD1 enzymes and abnormal production of ROS and LPO products, causing necrosis and death of the affected neurons in ALS; LPO-modified proteins are also associated with neural disruption in ALS. AD, Alzheimer’s disease; PD, Parkinson’s disease; ALS, amyotrophic lateral sclerosis; ROS, reactive oxygen species; NADPH, nicotinamide adenine dinucleotide phosphate; LOX, lipoxygenase; LPO, Lipid peroxidation; SOD1, superoxide dismutase 1.

Neurodegenerative diseases is a collective term for the clinical conditions characterized by gradual and progressive severe damage to neuronal cells, particularly in the central nervous system (CNS), which results in the loss of functions that are associated with the affected brain region [1,2]. Different in etiology and clinical symptomatology, NDDs share some common features at the cellular and molecular levels, such as protein misfolding, aggregation, and deposition; mitochondrial disfunction; chronic inflammation; and, oxidative damage of biomolecules, including lipids, proteins, and DNA (Figure 1). The most common type of NDD is Alzheimer’s disease (AD), accounting for approximately two-thirds of all cases [3]. Other NDDs include Parkinson’s disease (PD), Huntington’s disease, multiple sclerosis, amyotrophic lateral sclerosis (ALS), and many other rare conditions, such as: prion diseases, motor neuron diseases, spinocerebellar ataxia, spinal muscular atrophy, Friedreich’s ataxia, and Lewy body disease. All of these diseases, whether genetic or acquired, lead to the progressive decline or even the complete loss of sensory, motor, and cognitive function. AD, PD, and ALS are typically found in the elderly and are primarily classified as proteinopathies, meaning that they are associated with the aggregation and deposition of misfolded proteins that trigger neurotoxicity through cellular stress pathways [4,5]. In ALS, both upper and lower motor neurons are affected [6]. The disruption of proteostasis (protein homeostasis) can occur at any step of protein synthesis, including during transcription, translation, and post-translational modification.

Chaperons, which are proteins that facilitate the formation of correct and stable protein conformations, recognition and translocation of misfolded proteins into the cytosol, and cooperation with the ubiquitin/proteasome pathway (UPP) or the autophagy–lysosome pathway (ALP) to trigger degradation of misfolded proteins, regulate protein folding [7,8]. The mammalian targets of the rapamycin (mTOR) and sirtuin (SIRT) signaling pathways are key regulators of clearance mechanisms to prevent accumulation of misfolded proteins in neurons. mTOR regulates the ALP, which destroys transient proteins in the cytoplasm and core organelles, whereas sirtuin, specifically SIRT1, regulates the UPP [9].

The accumulation and aggregation of misfolded proteins in the brain and tissue, i.e., amyloid ß-peptide (Aß) in AD, α-synuclein in PD, ubiquitinated proteins in ALS, and their spread from cell to cell significantly contribute to the progression of NDDs [10]. The aggregation of these proteins in the endoplasmic reticulum (ER), a condition that is referred to as ER stress, activates a group of transcriptional signaling molecules, called the unfolded protein response, which aims to clear unfolded proteins, restore ER homeostasis, and ensure cell survival. In ER stress, reactive oxygen species (ROS) are generated, leading to chronic oxidative stress [11]. The accumulation of misfolded proteins in mitochondria also leads to their disfunction. Mitochondria play important roles in cell respiratory processes, metabolism, intracellular signaling, free radical production, apoptosis, and adenosine triphosphate (ATP) synthesis through oxidative phosphorylation. Mitochondrial disfunction leads to the increased production of ROS and oxidative phosphorylation defects and plays pivotal roles in ageing and the pathogenesis of NDDs, as neurons are especially vulnerable and susceptible to oxidative stress, because of their high energy requirements and high oxygen turnover [12].

Oxidative stress biomarkers have been developed due to the interaction of reactive oxygen and nitrogen species with major biomolecules, like carbohydrate, lipids, proteins, and nucleic acid. Besides the abovementioned cellular enzymes, endogenous ROS production is induced by the actions of lipoxygenase, myeloperoxidase, angiotensin II, and nicotinamide adenine dinucleotide phosphate (NADPH) oxidase. Naturally, antioxidant enzymatic and non-enzymatic defense factors maintain a balance and alleviate oxidative stress. The main components of enzymatic antioxidant defense include superoxide dismutase, glutathione peroxidase and reductase, glutamyl transpeptidase, and catalase, whereas non-enzymatic endogenous antioxidants include glutathione (GSH), uric acid, ubiquinone, tocopherol, retinol, melatonin, and nuclear factor erythroid 2-related factor 2. In NDD, ROS production is increased and the defense system is weakened, which further aggravates the condition [13,14,15].

The onset of NDD is associated with a decline in autophagy activity, i.e., the incorporation of cargoes, such as proteins, organelles, and microbial invaders, into autophagosomes, as a result of genetic variation, ageing, or lifestyle [16]. Autophagosome formation and maturation depends on available lipids and lipid-binding proteins, thus indicating the significance of lipids in neural tissue health and pathology.

## 2. Lipids in the Central Nervous System (CNS)

Evidence is emerging that, besides disturbances in protein metabolism, the disturbances in lipid metabolism, particularly of phosphoinositols and sphingolipids, also play significant roles in neurodegeneration.

Lipids are key components of the structural and functional organization of the CNS, composing almost 60% of the dry mass of human brain. Lipid properties and the effects in the CNS are directly determined by the proportion of specific fatty acids in their molecular structure and, in particular, by the content of long-chain polyunsaturated fatty acids (PUFAs). PUFAs represent about 35% of total brain lipids and are mostly bonded in phospholipids; palmitic acid (16:0), stearic acid (18:0), and oleic acid (18:1n-9) together account for ~50% of the total, while all of the other fatty acids constitute less than 20% of the human brain [17,18]. The most abundant PUFAs in brain tissue are those that belong to the omega-3 (n-3) and omega-6 (n-6) series: arachidonic acid (20:4n-6; AA) and docosahexaenoic acid (22:6n-3; DHA) [19]. AA and DHA can both be produced from their precursors through the activity of the Δ5- and Δ6-desaturase and elongase in liver cell and brain cell endoplasmic reticulum and peroxisomes. Humans are relatively inefficient in performing this synthesis [20]. Thus, the majority of AA and DHA have to be consumed in the diet.

The three basic mechanisms of PUFA effects on the nervous system are: (1) modulation of the physical properties of the cell membrane, (2) secondary messenger activity, and (3) regulation of gene expression [21]. The presence of PUFAs in neural phospholipids favorably affects membrane permeability and fluidity, and it promotes endo- and exocytosis, ion channel activity, and activity of membrane-bound proteins including neurotransmitter receptors [19]. The effects of PUFAs on secondary messenger activity are related to the action of enzyme phospholipases. Phospholipases act directly on the membrane structure, liberate PUFAs from membrane phospholipids, and generate free fatty acids, which can serve as endogenous secondary messengers. In this way, AA that is released from the nerve cell membrane by the enzyme phospholipase A2 participates in the transmission of the signals responsible for the growth, activity, and maturation of neural branches into mature synaptic terminals [22]. PUFAs regulate gene expression by direct binding to the gene transcription factors or after being translated into biologically active compounds, such as eicosanoids and prostaglandins [23]. The products of the genes that are activated by AA and DHA participate in the interaction of nerve cells, enter the membrane ion channels, and contribute to neuro- and synaptogenesis [24,25]. DHA plays important roles in the regulation of the genes responsible for the glial response to CNS injury, as well as in the inhibition of proinflammatory and proapoptotic genes and the stimulation of antiapoptotic genes [26].

The amount of PUFAs, particularly n-3 PUFAs, in the brain decreases during ageing [27,28]. The n-3 PUFAs content of the aged brain largely depends on n-3 PUFAs intake during the life span. However, reduced activity of key enzymes involved in the biosynthesis of long-chain n-3 PUFAs from dietary precursors, Δ6- and Δ5-desaturases, is also found in the aged brain. Normal ageing is connected with decreased antioxidant capacity, an increased rate of lipid peroxidation (LPO), and the consequent decrease in n-3 PUFAs in brain tissue [29], which results in the altered chemical composition, structure, and function of the aged brain [19]. Johnson et al. reported a decrease in antioxidants and an increase in LPO in elderly people when compared with young adult controls [30]. Several reports confirmed the age-related weakening of the enzymatic antioxidant defense [31]. Moreover, lipid hydroperoxide and thiobarbituric acid-reactive substances have been identified as sensitive markers of normal ageing [32]. Therefore, LPO is associated with the development and progression of NDDs as well as with normal ageing.

## 3. Lipid Peroxidation

LPO is a complex non-enzymatic process that occurs in three distinct stages: initiation, propagation, and termination. The process is initiated when reactive oxygen metabolites cause hydrogen abstraction from the methylene group of the carbon–carbon double bond of PUFAs molecules, thus forming a fatty acid radical. These unstable compounds stabilize their molecular structure by forming conjugated dienes with the concomitant production of carbon-centered alkyl radicals [33]. The oxidation of the carbon-centered alkyl radical with para-magnetic molecular oxygen generates a lipid peroxyl radical that subsequently attacks another PUFAs. In the propagation stage, the process continues as an uncontrolled self-perpetuating chain reaction, which leads to the amplification of the initial oxidative event. Potentially, all PUFAs in the membrane might be oxidized [34]. Termination occurs when different types of radicals react mutually to form stable products, when radicals react with chain-breaking antioxidants (e.g., vitamin E) and produce non-radical products, or when the substrate is depleted [35]. In addition, the generation of LPO toxic products can be significantly decreased by the activity of antioxidant enzymes, such as catalase (CAT), superoxide dismutase 1 and 2 (SOD1 and SOD2, respectively), peroxiredoxin (Prx), glutathione peroxidase (GPx), glutathione reductase (GR), and heme oxygenase-1 (HO-1) [36,37].

## 4. Products of LPO

The primary products of LPO are unstable peroxides or hydroperoxides that can be further degraded to secondary products, including hydrocarbons, alcohols, ether, epoxides, and aldehydes. They react with proteins, lipids, nucleic acids, cofactors, and vitamins, thus influencing their structure and function [38,39]. Linoleic acid (LA), arachidonic acid (AA), and docosahexaenoic acid (DHA) are the fatty acids that are most commonly oxidized in the brain [40].

### 4.1. Oxidation Products from LA

**Figure 2:**
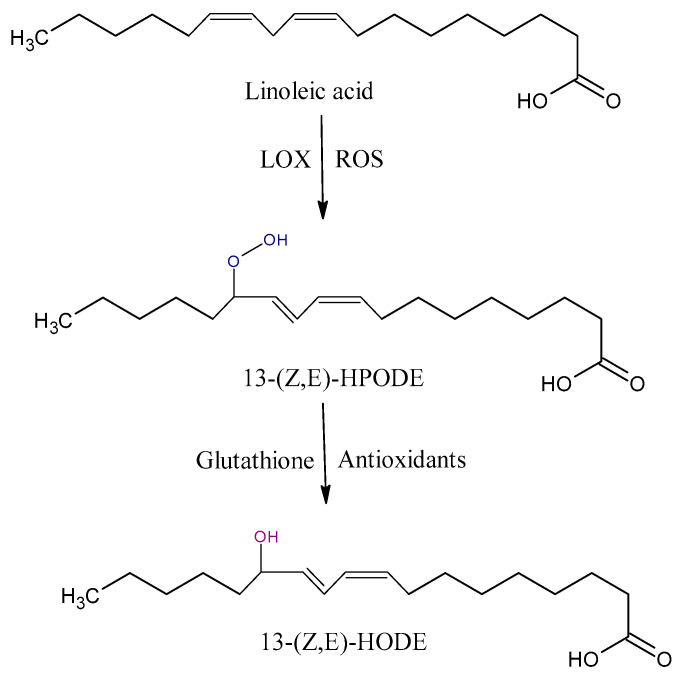
Some of oxidation products from linoleic acid (LA). HPODE, hydroperoxyoctadecadienoic acid; HODE, hydroxyoctadecadienoic acid; LOX, lipoxygenase.

Hydroperoxyoctadecadienoic acids (HPODE) are formed by radical-mediated and/or enzymatic oxidation via the lipoxygenase (LOX) of linoleic acid (LA). They exist as four isomers, 13-(*E*,*E*)-HPODE, 9-(*E*,*E*)-HPODE, 13-(*Z*,*E*)-HPODE, and 9-(*Z*,*E*)-HPODE, which can be further reduced to hydroxyoctadecadienoic acid (HODE) by glutathione peroxidase (Figure 2) [35]. 13-HODE may generate an anti-inflammatory 13-octadecadienoic acid (13-oxoODE) in a dehydrogenation process that is mediated by NADPH-dependent fatty acid dehydrogenases [41]. The biosyntheses of 13-HPODE and HODE are associated with the pathology of severe inflammatory based diseases [42,43]. 13-HODE incorporates into phospholipids and neutral lipids; as a major component of oxidized low-density lipoprotein (LDL) and atherosclerotic plaques, it plays a central role in the pathogenesis of atherosclerosis [44].

### 4.2. Oxidation Products from AA

**Figure 3:**
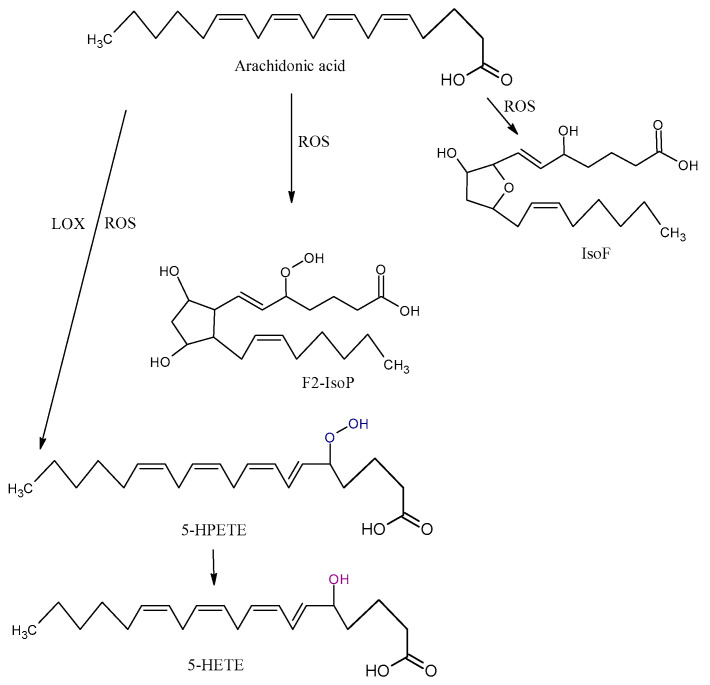
Three types of oxidation products of arachidonic acid (AA): isofurans (IsoFs), isoprostanes (IsoP), hydroperoxyeicosatetraenoic acid (HPETE), hydroxyeicosatetraenoic acid (HETE), ROS, and LOX.

Arachidonic acid is vulnerable to free-radical-mediated oxidation. As consequences of this process, six hydroperoxyeicosatetraenoic acid products (5-, 8-, 9-, 11-, 12-, and 15-hydroperoxyeicosatetraenoic acid (HPETE)) are formed (Figure 3). Some of them may be generated by LOX enzymes [45]. All the HPETE molecules may be further reduced, generating hydroxyeicosatetraenoic acid (HETE). Literature data indicate that 15-HETE incorporates into phospholipids, especially phosphatidylinositol, and thus changes its structure and function and indirectly influences signal transduction in the cells [46]. 20-HETE is another product of AA oxidation that is formed by mediation cytochrome P450 oxidoreductase.

The isoprostanes (IsoPs) F, E, and D are a series of prostaglandin-like compounds formed via free-radical-initiated peroxidation of AA. As chemically very stable molecules, F2-IsoPs are considered to be the most reliable markers of oxidative damage in humans [47]. F2-IsoPs appear in four isomers, 5-, 12-, 8-, and 15-series, each of which comprises eight diastereomers, forming a total of 64 compounds [48]. The formation of IsoPs affects membrane fluidity and integrity. In addition, IsoPs may be released from cell membranes by phospholipases; they then circulate in plasma, where they further affect other molecules. Several studies demonstrated that the level of isoprostanes is higher in some NDDs. Although IsoPs are mainly generated from AA, F-ring IsoPs may be formed from the peroxidation of other PUFAs with three double bonds, such as alpha-linolenic acid, EPA, and DHA.

The oxidation of AA also leads to the formation of isofurans (IsoFs). IsoFs, as biomarkers of oxidative stress, are used in clinical settings when high concentrations of oxygen are used during treatments or procedures [40].

### 4.3. Oxidation Products from DHA

**Figure 4:**
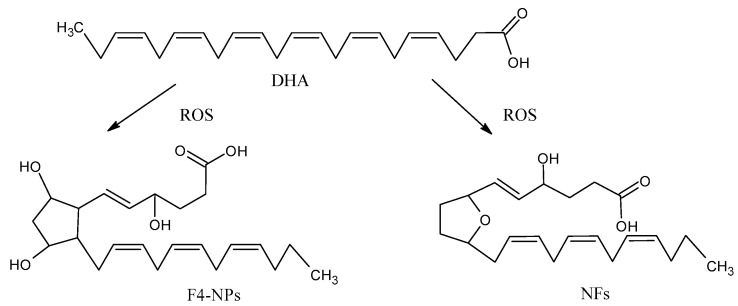
Oxidation products from docosahecsaenoic acid (DHA): neurofurans (NFs) and neuroprostanes (NPs).

The peroxidation of DHA generates eight isomers with a total of 128 compounds named neuroprostanes (NPs) (Figure 4), which are abundantly concentrated in the neuronal membranes [49]. Although their biological roles are not entirely clear, some authors found that NPs have anti-inflammatory properties [50]. The neurofurans (NFs) are also oxidation products from DHA [51]. NPs and NFs are both sensitive and specific markers of neuronal oxidative damage, and their analysis could more accurately reflect the levels of lipid peroxidation in DHA-rich tissues, such as the brain.

### 4.4. Short-Chain Aldehydes

**Figure 5:**
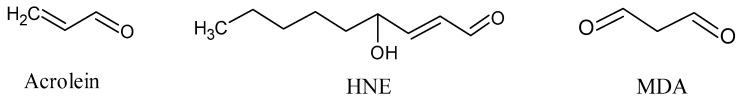
Short-chain aldehydes: acrolein, 4-hydroxy-2-hexenal (HNE), and malondialdehyde (MDA).

The final products of lipid peroxidation of PUFAs are reactive short-chain aldehydes (Figure 5) [52]. These short-chain aldehydes are mainly classified into three families: 2-alkenals, 4-hydroxy-2-alkenals, and ketoaldehydes. Among them, acrolein (2-alkenal) is the most reactive, whereas 4-hydroxy-2-nonenal (HNE) and malondialdehyde (MDA) are the most abundant [53].

Acrolein, which is an α, β-unsaturated aldehyde, is produced from the lipid peroxidation of PUFAs. As with other LPO peroxides, acrolein is capable of initiating a further process of lipid peroxidation: it attacks and deforms mitochondrial membranes, and then reacts with DNA and proteins [54]. Acrolein induces non-programmed necrosis and apoptosis [55], modification and aggregation of the protein, and inactivation of enzymes [56]. Generally, acrolein is capable of inducing cellular degeneration and death, and particularly the deterioration of hippocampal neurons [57]. In vitro studies have demonstrated the neurotoxic effects of acrolein on different types of cells line, thus confirming that acrolein plays a neurotoxic role in CNS neurodegeneration [58].

4-hydroxy-2-alkenal (HNE) is formed by peroxidation of n-6 PUFA, especially LA and AA, as a product of a non-enzymatic process in which an initial hydroperoxide undergoes fragmentation and form HNE [59]. n-3 PUFA, especially alpha-linoleic acid, may be attacked by free radicals, and its peroxidation generates 4-hydroxy-2-hexenal (HHE). Although HNE usually remains associated with the site where it is generated, HNE can diffuse to different cellular compartments and interact with many different substrates, including covalent binding to cysteine, histidine, and lysine residues [60]. Some plasma membrane ion and nutrient transporters, such as Na^+^/K^+^-ATPase, glucose, and glutamate transporters, several receptors for growth factors, neurotransmitters, mitochondrial, cytoskeletal, and proteasomal proteins, as well as proteins that repair oxidative damage may be targets for HNE [61]. As a signal molecule, HNE may suppress the activity of nuclear transcription factor κB [62] and activate the caspase pathways, leading to cell death in NDDs [63].

Malondialdehyde (MDA), a ketoaldehyde, which is an extremely reactive and toxic aldehyde, is generated by the decomposition of AA and larger PUFAs through enzymatic or non-enzymatic processes [64]. MDA can be generated during the enzymatic biosynthesis of thromboxane A2 [65]. It often has a relatively longer half-life and may, therefore, diffuse from the places of generation to other sites in vivo, further increasing oxidative and carbonyl stresses. MDA is capable of interacting or crosslinking on cellular and tissue proteins or DNA, resulting in the formation of adducts and biomolecular damage [66].

## 5. NDDs, LPO, and Antioxidants

Evidence shows that the high incidence of NDD may be attributed in part to the negative influence of daily risk factors, including stress, lack of physical exercise, and unhealthy nutrition. As such, as oxidative stress plays a crucial role in the process of neurodegeneration, numerous studies have documented the beneficial effects of exogenous nutritional antioxidants as neuroprotectors [67]. Nutritional antioxidants can modify oxidative stress on several levels: by decreasing the production of ROS and repairing the oxidized membranes, by neutralizing the free radicals, or through lipid metabolism in which cholesteryl esters and short-chain free fatty acids neutralize ROS [68]. Fruits, vegetables, beverages, green tea, coffee, spices, nuts, and cereal products are major sources of plant-derived antioxidants: polyphenols (phenolic acids, flavonoids, anthocyanins, lignans, and stilbenes), carotenoids (xanthophylls and carotenes), and vitamins (vitamins C and E) [69]. Table 1 summarizes the beneficial effects of the plant-derived antioxidants on NDD.

## 6. Alzheimer’s Disease

Alzheimer’s disease is a progressive brain disorder that is associated with neuronal degeneration and a loss of neurons in brain regions controlling memory, cognition, and emotional behaviors. AD patients experience rapid declines in the ability to learn, reason, maintain emotional stability, communicate, and perform common daily functions [101]. The onset of familial AD relates to genetic mutations in enzymes that are involved in amyloid precursor protein (APP) processing, whereas the etiology of sporadic AD is still unclear [102]. The complex and heterogeneous AD pathophysiology is dominated by two main hallmarks: overproduction and extracellular deposition of Aß and the formation of intracellular neurofibrillary tangles (NFTs), consisting of hyperphosphorylated tau protein [103]. The aberrant aggregations of Aβ and tau create an overall cytotoxic environment that results in the disturbance of neuronal cell shape and function, including the disturbance of ATP production, axonal transport, and synaptic signaling, together leading to severe cognitive and motor impairment characteristic for AD [104].

LPO is an important factor in the pathogenesis of AD (Figure 1) [105]. Initially, the oxidative stress environment that prevails in the AD brain induces LPO, which, in turn, further promotes disturbance of antioxidant capacity within the brain cells [106]. The elevated levels of LPO products and LPO-modified proteins, all of which are recognized as neurotoxic agents, have been found in AD-affected subjects. HNE occurs in several NDDs, such as AD, PD, ALS, Huntington disease, and Down syndrome [107,108,109,110]. Increased levels of HNE–protein adducts have been detected in the diseased brain regions and body fluids of AD patients. HNE commonly targets proteins involved in energy metabolism and the antioxidant response, leading to the disturbance of these important cell functions and, consequently, to neuronal dysfunction and death [111]. In addition, HNE–protein adducts add to the stimulation of the autoimmune response [112]. IsoPs and NPs are also increased in AD [113]. High levels of F2-IsoP were found in the hippocampus and the cerebrospinal fluid (CSF) of patients with AD [114]. Acrolein, which is predominantly localized within NFTs, directly attacks DNA, reacts with DNA base guanine, and forms acrolein–deoxyguanosine, which are excessively presented in the AD brain [101,113]. There are clinical studies reporting neurotoxicity due to increased acrolein levels in the brain and spinal cord of patients with AD and spinal cord injury [115,116]. An elevated level of circulating HODE, which is released from phospholipids by phospholipases, has been found in the plasma and erythrocytes of patients with AD [117]. However, MDA is the most abundant LPO product [118]. Significantly higher levels of MDA are found in AD patients when compared to healthy subjects [119]. MDA accumulated in the AD brain covalently binds to a variety of proteins and promotes the formation of aberrant protein adducts, which disturb nerve signaling and structure in AD-affected brain regions, such as the frontal, temporal, and occipital lobes and hippocampus [120].

### AD and Antioxidants

Because oxidative stress and LPO lie at the basis of neuronal damage in AD, the potential benefits from dietary supplementation with antioxidants, such as polyphenols, and antioxidant vitamins tocopherol (vitamin E), ascorbic acid (vitamin C), and carotenoids (vitamin A), have become the subject of considerable scientific interest. The majority of studies were conducted in animal models and in vitro; reports on the effects of antioxidant supplementation in humans with AD are sparse (Table 1).

Polyphenols are phytochemicals that are widely present in plant drinks and foods [121]. Because of their small molecules and lipophilic nature, they can cross the blood-brain barrier and exert strong antioxidant and radical scavenging activity within the brain tissue [122,123]. Among others, the most investigated polyphenols are curcumin, epigallocatechin gallate (EGCG), and rosmarinic acid, which exert many beneficial effects on AD pathology, although the majority of the results were produced from studies that were conducted on animal models and in vitro [124,125]. In a mice model of familial AD, EGCG significantly decreased Aß production and induced marked increases in brain synapses; these effects were accompanied by improved spatial learning and memory [126,127]. A few human studies investigating supplementation with curcumin, a polyphenol from turmeric, have found improved cognitive status in AD patients [80,128]. The consumption of EGCG-rich green tea correlated with decreased risk of neurodegeneration and cognitive impairment [129,130], whereas resveratrol, a polyphenol from red wine, increased brain blood flow and oxygen uptake, and improved auditory, verbal, and learning memory in AD patients [79]. Similarly, pills containing polyphenols from blueberry and green tea increased cognitive processing in treated older adults when compared to placebo [131]. Mastroiacovo et al. reported improvement in cognitive function in the elderly due to regular cocoa flavanols consumption, which is in line with results of Nurk et al., who reported increases in the cognitive abilities in the elderly consuming a diet high in flavonoids-rich food, such as wine, chocolate, and tea in a dose-dependent manner [130,132]. The protective effects on cognition were also found in people that consumed a diet based on walnuts, which are rich in polyphenols [133].

In patients with MCI, supplementation with vitamin E slowed disease progression and reduced risk of dementia [90]. Parallel supplementation with vitamin C has been shown to improve the beneficial effect of vitamin E, leading to a decrease in AD incidence and prevalence [134,135]. In contrast, other groups reported no benefits of vitamin E in patients with MCI [87], or in the prevention of AD, even in combination with selenium [89]. Vitamin E overdose correlated with an increased risk of mortality [136]. Various in vivo and in vitro studies reported decreases in oxidative stress and Aß peptide oligomerization by vitamin C supplementation. Studies in humans also found reduced oxidative stress, systemic inflammation, and atherosclerosis in persons that were supplemented with vitamin C [137,138]. However, randomized clinical trials still failed to demonstrate any association between vitamin C and alleviation of AD pathology, which indicated that the prevention of deficiency seems to be more beneficial than vitamin C supplementation [139]. The in vitro results for vitamin A indicated reduction in Aβ plaques and a decrease in cognitive impairment due to vitamin A and β-carotene supplementation [140,141]. In AD patients, high levels of these vitamins are associated with better memory and learning performance.

## 7. Parkinson’s Disease

Parkinson’s disease is the second most common neurodegenerative disorder, with incidence being consistently higher in men than in women that increases over the age of 60 years [142]. The most common clinical manifestations in patients with PD are resting tremors, slowness of movement, rigidity, and postural instability, along with other symptoms, such as dementia, depression, insomnia, and anosmia. As a chronic and progressive neurodegenerative disorder, PD is associated with an increased turnover of dopamine and reduced levels of striatal dopamine and its metabolites in the brain (Figure 1). The persistent and diffuse degeneration of dopamine-producing neurons in the substantia nigra pars compacta (SNpc) is observed. The pathogenesis of PD is characterized by misfolding and aggregation of proteins, particularly small synaptic protein α-synuclein, which is the main component of Lewy bodies [143]. α-synuclein was found to aggregate and accumulate in the remaining neurons in the SNpc, locus coeruleus, cerebrospinal cord, enteric nervous system, and autonomic ganglia [144]. The role of α-synuclein is primarily connected with the effects on mitochondrial processes and the formation of synaptic vesicles [143]. It has been proven that reduced levels of glutathione, an antioxidant critical for protecting dopaminergic neurons in the SNpc from free radical damage, increase LPO, which is involved in the pathogenesis and progression of PD [145].

Clinical studies have reported significant acrolein levels in the brain. Acrolein promotes initiation of LPO and further elevation of oxidative stress, as indicated by acrolein-induced increases in HNE [146]. Additionally, acrolein acts on the modification of α-synuclein in dopaminergic neurons, leading to mitochondrial dysfunction [147]. This results in ROS-mediated apoptosis of the affected neurons [148]. There are three independent mutations in α-synuclein, including A53T, A30P, and E46K, which are involved in the development of familial PD. However, mutation and aggregation of α-synuclein can both cause Parkinsonism. The misfolded α-synuclein protein is soluble and it serves as a mediator of neurotoxicity in dopaminergic neurons. Clinically, the accumulation of acrolein-α-synuclein adducts was detected in the nigral dopaminergic neurons of PD patients [149], in parallel with results from in vivo studies that found that acrolein acts as a Parkinsonian neurotoxin in the nigrostriatal dopaminergic system of rat brain [58]. In addition, the role of DHA in α-synuclein oligomerization and aggregation has been suggested [113,150].

4-HNE and Nε-(carboxymethyl) lysine have been localized in Lewy bodies in post-mortem PD brain tissue [151]. HNE modification leads to conformational changes and the oligomerization of α-synuclein. The modified oligomers are toxic and may contribute to the deterioration of neurons [152]. It was found that HNE modifies the transport and possibly the loss of dopamine since its content increases proportionally to the severity stages of PD [153]. Elevated HNE, protein accumulation, and dopamine loss probably affect the physical capabilities and the process of learning in patients with PD [147]. Another actin-binding protein has been observed in cell lines and it acts to regulate the development of the actin microfilament [147]. Upon LPO, this protein decreased in the PD patients, which led to the reduced recovery of dynamic development of neurons [154]. Consequently, this results in the muscle damage widely observed in patients with PD. LPO grades are higher in PD patients; plasma levels of F2-IsoPs, HETEs, 7β-hydroxycholesterol (7β-OHCh), and 27-hydoroxycholesterol (27-OHCh) 7-ketocholesterol (7-KCh), and NPs are higher when compared to healthy subjects [155]. In particular, plasma F2-IsoPs and HETEs levels are elevated in the early stages of PD. Interestingly, IsoFs but not F2-IsoPs are increased in the SNpc of patients with PD [156]. Elevated levels of HNE, MDA, and acrolein have been reported in Lewy bodies in the brain stem and neocortical neurons, as well as in the CSF of living PD patients [147,148,154].

### PD and Antioxidants

Evidence is increasing that oxidative stress plays a role in the pathogenesis of PD [157], and supplementation with the abovementioned antioxidants could produce beneficial effects in PD patients (Table 1).

Agents such as flavonoids that can target ROS and mitochondrial dysfunction are prime candidates for neuroprotection in PD [158,159]. The Mediterranean diet is a rich source of antioxidant bioflavonoids and polyphenols, which are associated with a decreased risk of PD [160,161]. Nevertheless, data on the polyphenol intake and the PD risk are contradictory. A large epidemiological study has shown that men with the highest quintile of flavonoid consumption (tea, berry fruits, apples, red wine, and orange or orange juice) had a 40% lower risk of developing PD when compared to those in the lowest quintile, but this was not observed in women [162]. However, 41-year follow-up Finnish data reported the association of berry consumption with increased risk of PD in men [163]. Other studies demonstrated that the consumption of green and black tea had beneficial effects in reducing the risk of PD [157,164]. In a rotenone model of PD, contrary to the previously reported neuroprotective effects that were observed in AD [165], pomegranate juice exacerbated oxidative stress and neurodegeneration [166]. Polyphenols, like curcumin, resveratrol, catechin, and oleuropein, inhibit the formation of Lewy bodies [167]. Overall, the preclinical and epidemiological data strongly support the further investigation of specific flavonoids for the treatment of PD [158].

The roles of vitamins in PD prevention and therapy are yet to be determined. So far, no link between vitamin A and PD has been established in few human studies [93,168,169,170], which was supported by a recent meta-analysis [170]. Only three case-control studies reported a significant association between lutein intake and PD risk, whereas two studies found a protective effect of dietary β-carotene intake [84,93] and risk of PD, one only in women [93]. The serum levels of some carotenoids—α-carotene, β-carotene, and lycopene—were lower in the PD patients, with evidence that carotenoids are inversely correlated with clinical variables that represent disease progression [84,171]. Decreased serum carotenoid levels are associated with poorer motor function [171]. 

As for vitamin A, despite numerous studies, no clear association between vitamin C and human PD [81,93,169,172] has been established. One study indicated that higher intake of fruits and vegetables containing vitamin C is associated with an increased risk of PD [173]. In contrast, in a case-controlled study, individuals consuming a diet rich in vitamin C showed a 40% reduction in PD risk [174]. It was suggested that supplementation with vitamin C may not affect disease development, because access to the brain is limited by high water solubility and the requirement for active transport at the choroid plexus to enter the brain [175]. The serum level of vitamin C in patients with PD remains controversial [176,177]. For instance, the vitamin C level in lymphocytes is significantly lower in patients with severe PD [178], suggesting that vitamin C supplementation may be beneficial for the treatment of PD. This was evident in a large cohort study, including patients with PD, which found that dietary vitamin C intake significantly reduced the risk of PD, but this effect was invalid for the four-year-lag analysis [85].

Based on literature data, among antioxidant vitamins, only vitamin E intake was found to be associated with a reduced risk of PD in three of the four studies [179]. A meta-analysis showed a protective effect against PD in humans with both moderate and high intake of vitamin E [169]. This effect is more pronounced in men than in women [94], and only higher vitamin E intake (>9.759 mg/day) is significantly associated with decreased risk of PD in women [93]. These protective effects may be achieved through preventing oxidative stress in cells and inhibiting apoptosis. Apart from PD risk, vitamin E has also been used in intervention studies, as PD patients were found to have lower serum levels of vitamin E than controls [180]. A high-dose supplementation (2000 IU/day) can significantly elevate the vitamin E level in CSF [92]. Vitamin E supplementation was tested as a therapeutic against PD in the Deprenyl and Tocopherol Antioxidative Therapy of Parkinsonism (DATATOP) study [181]. However, no beneficial effect of α-tocopherol was observed during follow-up evaluation of PD symptoms [181]. Recently published data indicate that vitamin E represents a potential therapeutic target for disease-modifying treatments in PD as a result of both the clinical retrospective analysis and electrophysiological experiments [88]. Additional trials are still needed in order to confirm the role of vitamin E in slowing the progressive deterioration of function in PD.

To summarize, a research gap exists in the effect of antioxidants supplementation on lipid peroxidation products in PD patients [182,183]. Data on supplementation in patients already diagnosed with PD has failed to show a disease-modifying effect.

## 8. Amyotrophic Lateral Sclerosis

Amyotrophic lateral sclerosis is an NDD that is characterized by progressive loss of motor neurons in the CNS, leading to muscular atrophy, paralysis, and death [184,185] ALS occurs in the sporadic form (sALS) in 90% of cases, and in the familial form due to inherited genetic mutations (fALS) with an estimated incidence of between one and two per 100,000 people [186]. Among all ALS patients, 50% die within 30 months of symptom onset, often from respiratory insufficiency, whereas about 10% of patients may survive for more than a decade [187,188]. Riluzole, which is the only Food and Drug Administration (FDA)-approved drug that is currently accessible for ALS, slows disease progression, and improves limb function and muscle strength, but, unfortunately, it increases life span by only 2–3 months [189]. The mean age at onset this disease is 40–60 years for fALS and 58–63 years for sALS, with a peak incidence at age 70–79 years [190]. Men have a higher risk of ALS than women, leading to a male-to-female ratio of 1.2–1.5 [191]. Genetic studies have shown that *C9orf72*, *SOD1*, *TARDBP*, and *FUS* are the most common mutated genes in ALS [192]. The first pathological mutation was identified in ALS patients in the *SOD1* gene in 1993 [193]. To date, over 180 different mutations have been described in the *SOD1* gene, which can be found in 10–20% of familial ALS cases and 1–5% of sporadic ALS [192,194]. The *SOD1* gene encodes the Cu/Zn SOD1, which is one to three isoenzymes of SOD responsible for the conversion of the superoxide anion radical to molecular oxygen and hydrogen peroxide. Mutations in *SOD1* lead to numerous alterations in the structure and function of motor neurons in ALS patients (Figure 1). Thus, the mutated enzymes result in misfolded protein chains, and they form small neurotoxic aggregates in the nuclei of glial cells (mostly astrocytes) of the spinal cord, which contributes to neuron degeneration [195]. The SOD enzyme may be post-translationally modified and hyper-oxidized in sALS patients; through this oxidation, altered SOD1 gains toxic properties [196]. In addition, the SOD mutant has reduced enzymatic activity, which results in an abnormal production of ROS, which causes an alteration in the cell function, apoptosis, and necrosis [197]. Previous pathological studies have reported evidence of increased LPO products in biological fluids of ALS patients compared with control samples. Several literature data demonstrate a significant increase in MDA leve-ls in the sera in ALS patients [198,199], thus strengthening the clinical evidence that prooxidative imbalances contribute to ALS pathophysiology [200]. In ALS, HNE is bound to three key proteins: dihydropyrimidinase-related protein 2 (DRP-2), heat shock protein 70, and α-enolase, which leads to their modification [201]. They are involved in axonal development, transmission, and modulation of extracellular signals; repair mechanism; and, maintaining of redox-homeostasis; thus, their modification leads to the loss of motor neuron function [53]. The elevated levels of HNE and HNE-modified proteins are observed in the spinal cord motor neurons of ALS patients [202]. In addition, when HNE attacks DNA, it may cause cellular damage and apoptosis [203]. Literature data demonstrate significantly elevated levels of HNE in the sera and spinal fluid of sALS patients when compared with control subjects, which were positively correlated with the extent of disease but not a rate of progression [204]. Moreover, these authors showed that HNE levels from sALS serum and CSF samples were significantly above those that were collected from fALS patients, suggesting that the familial and sporadic forms are qualitatively different concerning oxidative stress. In accordance, the same group of authors previously documented increased levels of HNE in the CSF and spinal cord motor neurons of ALS patients [109,202]. Several studies examined the level of IsoP in patients with ALS. Significantly higher levels of F2-IsoPs have been found in the urine of patients with sALS when compared with healthy subjects [205]. However, Montine et al. did not find differences in IsoP among ALS patients and healthy subjects [114].

### ALS and Antioxidants

Evidence shows that neural oxidative damage contributes to neuronal oxidation, dysfunction, and degeneration in ALS, as discussed above. In line with this, inhibition or suppression of neuronal oxidation may slow or even stop disease progression. Among natural antioxidants, vitamins A, E, and C, as well as polyphenols-rich fruits, can be potential antioxidants whose effect could be investigated. However, the literature data about the effects of antioxidative therapy in ALS are scarce (Table 1) and they are mostly limited to in vitro studies and experimental model studies.

Despite a well-documented protective role against LPO [206], only a few studies examined the effect of vitamin E on ALS. One of them investigated the influence of vitamin E supplementation on survival and motor function in ALS. After three months of treatment with vitamin E (500 mg twice daily) and riluzole as standard drug therapy in ALS, a decrease in plasma MDA levels and an increase in plasma GSH levels were observed [96]. However, survival was not influenced by the treatment. Additionally, Ascherio et al. observed that regular use of vitamin E supplements for 10 years or more was associated with a lower risk of dying from ALS [97]. Similarly, Wang et al. found a positive trend for a decline in ALS progression in patients with long-term use of vitamin E supplements, but did not find an overall protective role for vitamin E [98]. In a randomized controlled trial (RCT) that was conducted in ex-Yugoslavia, a combination of methionine, vitamin E, and selenium led to a significant increase in the rate of survival and increase in activity of GPx in 28 patients with ALS after 12 months of supplementation [99]. However, the analysis of data from 10 different RCT studies with 1050 participants and shorter durations of the supplementation period revealed no significant beneficial effect of vitamin E on survival in ALS patients [207]. Studies on the impact of vitamin C on progression and duration of ALS are also rare. A recently-published original study, including 202 patients with ALS, showed lower serum level of vitamin C level in patients with ALS when compared to healthy controls [208]. Conversely, in a study with only 19 ALS patients, there was no significant difference in plasma and CSF vitamin C levels compared with controls [209]. The results from pooled analysis of five large prospective studies with 1093 cases of ALS indicated that high dietary intakes or supplemental use of vitamin C appear to not affect the risk of ALS [86]. One animal study indicated that a high dose of vitamin C administered before the onset of disease prolonged survival and motor function in fALS transgenic mice, whereas vitamin C that was administered after the onset disease did not have effect on survival [210].

A recently published study showed that the level of vitamin A is significantly higher in patients with ALS when compared to healthy controls [208]. Conversely, in a case-controlled study with 77 patients with ALS, β-carotene was found to decrease the risk of sporadic ALS [211]. Several studies have documented a beneficial association between ALS and the intake of carotenes [212,213], as well as the consumption of foods rich in carotenoids helping the prevention or even delay of the onset of ALS [86]. According to our knowledge, there are no data about the influence of vitamin A on the level of LPO and/or other parameters of oxidative stress in ALS patients. Additionally, long-term dietary supplementation with retinoic acid has been reported to shorten the lifespan in an ALS mouse model [214].

Flavonoids are among the natural substances that are present in fruits and vegetables and have a protective effect against ROS. However, few studies examined whether these molecules impact ALS. Among them, Korkmas et al. showed that chronic administration of 7,8-dihydroxyflavone significantly improved motor deficits and enhanced lower neuronal survival in the transgenic ALS mouse model [215], whereas Ip et al. found that quercetin and its derivative could be therapeutic inhibitors of the aggregation and misfolding of SOD1 that is noticed in ALS [216].

## 9. Future Perspectives

Searching the FDA registry, Cummings et al. found 121 pharmacologic agents currently being investigated in clinical trials for the treatment of AD. The majority of agents (17 out of 29) in 36 Phase III trials are disease modifiers that mainly target amyloid, inflammation/infection/immunity, and synaptic plasticity. Among them, gingko biloba extract, rich in flavonoids, is the only natural antioxidant [217]. Plant extracts may produce better antioxidant/disease-modifying activities than a single compound through the additive or synergistic effects of their different active ingredients and a variety of secondary metabolites [218]. Evidence is accumulating that combination of FDA-approved drugs with natural antioxidants, as well as combinations of appropriate antioxidants, may be more effective, considerably lower cost, and, therefore, more affordable and acceptable, especially for long-term prevention of NDDs [68,219].

Dietary habits and nutrition consumption affect cognitive functions. The peak of cognitive function is around 20 or 30 years of age, and then cognitive functions decline after 50 or 60 years of age. Evidence suggests that diet interventions show promise for dementia prevention [220]. Clinical trials for the prevention decline of cognitive function in adults with antioxidants are still in their infancy. Polyphenol berry fruit juice consumption was most beneficial for immediate verbal memory. Isoflavone-based interventions were associated with significant improvements for delayed spatial memory and executive function [221]. However, no clear evidence exists for an association between cognitive outcomes and polyphenol dose response, duration of intervention, or population studied. Therefore, further studies are needed in order to determine whether long-term antioxidants intake can reduce the risk of memory loss in adult population.

## 10. Conclusions

No cure exists for NDDs, particularly in advanced stages. The drugs that are approved by the FDA, such as acetylcholine esterase inhibitors (donepezil, rivastigmine) as well as levodopa for PD, which crosses the blood–brain barrier and restores dopamine levels in the substantia nigra, only ameliorate the symptoms and slow the progression of the diseases for several years [1]. Newer therapy approaches that are focused on neuroregeneration, i.e., structural and functional recovery of the damaged nervous system through immunomodulation, inhibition of formation of protein aggregates, disaggregation of misfolded proteins, and induction of autophagy, give hope that the degeneration process of afflicted neurons might be slowed and the recovery rates and longevity improved [222]. Because of the complex nature of NDDs, a multi-target drug approach is encouraged, as it may produce additional beneficial effects. When considering the role of lipid peroxidation in the development and progression of NDDs, further human studies applying antioxidant supplementation in different forms of NDD are urgently needed.

## Figures and Tables

**Table 1:** Dietary antioxidants intake and supplementation in prevention and adjuvant treatment in patients with neurodegenerative disease (NDD).

Antioxidant	Treatment	Population	Effect/Conclusion	References
	Doses/Exposure			
	Assessment			
**POLYPHENOLS**				
***Flavonoids***				
**Flavonols** (quercetin, kaempferol, myricetin): onion, broccoli, tomato, kale, celery, grapes, apple				
Grape seed extract	250 mg/day 12 weeks	111 elderly persons	Improves physiological cognitive profiles (attention, language, immediate and delayed memory)	Calapai et al. [70]
Flavonols-rich diet	Dietary estimated follow-up 6 years	921 elderly persons	Dietary flavonols inversely associated with incident AD	Holland et al. [71]
**Flavones** (luteolin, apigenin): parsley, celery, hot peppers, thyme				
Luteolin	Spalmitoylethanolamide combined with luteolin 700 mg/day 4 weeks	17 patients with FTD	Reduced behavioral disturbances and improved frontal lobe functions	Assogna et al. [72]
**Flavanones** (naringenin, hesperetin): grapefruit, orange, lemon, citrus peel, citrus fruits				
	Dietary citrus	13,373 elderly persons, follow-up 5–7 years	Lower risk of incident dementia	Zhang et al. [73]
**Anthocyanins** (cyanidin, malvidin, petunidin, peonidin): strawberries, raspberries, blueberries, bilberry, elderberry, black currant, other berries, cherries, pomegranate, red wine, red onions				
	30 mL/day blueberry concentrate (387 mg anthocyanidins) 12 weeks	12 older adults/ 8 placebo	Improvement in working memory	Bowtell et al. [74]
**Isoflavones** (genistein): soy, tofu				
	100 mg soy/day, 6 months	59 AD patients	No improvement in cognition	Gleason et al. [75]
	Soybean-rich diet	1006 elderly persons, follow-up 15 years	Reduced risk of dementia	Ozawa et al. [76]
	Japanese dietary pattern	14,402 older adults, follow-up 5–7 years	Decreased risk of incident dementia	Tomata et al. [77]
**Flavan-3-ols** (flavanols): green tea, red grapes, red wine				
Epigallocatechin-3-gallate (EGCG)	One gelatin capsule (400 mg epigallocatechin gallate) orally once per day for 4 weeks, then one capsule twice daily for 4 weeks, and then one capsule three times daily for 40 weeks, RCT	47 intervention, 45 controls with multiple system atrophy	Did not modify disease progression	Levin et al. [37]
	Green tea	2015 elderly persons	Associated with low prevalence of AD and severe cognitive impairment; modulated the CNS immune response	Yang et al. [78]
***Stilbenes***: red grapes, red cherries, peanut, pomegranate, berries				
Resveratrol	500 mg/day orally	119 patients with mild to moderate AD	Preserved the blood–brain barrier integrity	Sawda et al. [79]
***Curcuminoids***: turmeric				
Curcumin	1–4 g/day for 6 months	36 patients with AD	Slight improvement in cognitive function	Baum et al. [80]
	1 g/day for 6 weeks	60 patients with MDD	Lower HDRS-17	Sanmukhani et al. [81]
		40 patients with MCI	Improved verbal memory and attention	Small et al. [82]
**VITAMINS**				
Vitamin C	200 mg/day ascorbic acid	67 elderly PD patients	Can improve levodopa absorption in elderly	Nagayama et al. [83]
	Dietary estimated	1329 PD patients	Dietary vitamin C inversely associated with PD risk in women at borderline significant	Yang et al. [84]
	Dietary estimated	1036 PD patients	Dietary vitamin C has no relationship with PD risk	Hughes et al. [85]
	<400 mg/day, 400–700 mg/day, ≥700 mg/day	1,100,910 participants 1093 developed ALS	Vitamin C not associated with reduced risk of ALS	Fitzgerald et al. [86]
Vitamin E	I: Vitamin E 2000 IU/day II: donepezil 10 mg/day III: placebo for 3 years	212 MCI AD patients	No benefit on cognition impairment in AD	Petersen et al. [87]
	Dietary estimated	1329 PD patients	Inverse association between dietary vitamin E and PD in women	Yang et al. [84]
	Dietary estimated	100 PD patients 100 controls	Higher dietary vitamin E inversely associated with PD occurrence independently from age and sex	Schirinzi et al. [88]
	Vitamin E (400 IU/day) + selenium (200 μg/day)	7540 non-demented men (≥62 years)	No effect on AD prevention	Kryscio et al. [89]
	1000 IU/day for 6 months to 4 years	613 patients with mild to moderate AD	Reduced functional decline	Dysken et al. [90]
	Vitamin E 2000 IU/day and/or Deprenyl 10 mg/day	800 untreated PD patients	α− tocopherol did not improve clinical features in patients with PD	Parkinson Study Group (DATATOP study) [91]
	Vitamin E 2000 IU/day	18 vitamin E group 5 placebo group	α− tocopherol levels increased in cerebrospinal fluid	Vatassery et al. [92]
	Dietary estimated	249 PD patients 368 controls	Higher consumption of vitamin E reduced risk of PD	Miyake et al. [93]
	Dietary estimated	1036 patients with PD	No relationship with PD risk	Hughes et al. [85]
	Dietary estimated	Total 124,221 371 PD cases	Intaking foods containing more vitamin E can reduce the risk of PD	Zhang et al. [94]
	10 mg/day	5342 individuals 31 PD cases	Dietary vitamin E may reduce the risk of PD	de Rijk et al. [95]
	500 mg/day	288 patients with ALS 144 vitamin E+ riluzole 144 placebo + riluzole 12 months	Vitamin E did not affect survival and motor function in ALS	Desnuelle et al. [96]
	Regular use	957,740 individuals, 525 developed ALS 10 years	Vitamin E supplementation associated with a lower risk of dying of ALS	Ascherio et al. [97]
	Women (8.8 IU/day) Men (14.6 IU/day)	1,055,546 participants, 805 developed ALS	Long-term use of vitamin E supplements could be inversely associated with risk of ALS	Wang et al. [98]
		28 ALS patients 14 Alsemet 14 placebo 12 months	Alsemet (vitamin E + methionine + Se) increased the rate of survival in ALS patients	Stevic et al., 2001, [99]
**Carotenoids** (α-carotene, β-carotene, β-cryptoxanthin, lutein, zeaxanthin, lycopene): cantaloupe, pasta, corn, carrots, orange/yellow peppers, spinach, broccoli, sweet potato, tomato, fish, salmon, eggs				
Xanthophyll carotenoids plus omega-3 fatty acids	lutein:meso-zeaxanthin:zeaxanthin 10:10:2 mg/day plus 1 g/day of fish oil for 18 months	13 patients with AD	Slowed the progression of AD with functional benefits in memory, sight, and mood	Nolan et al. [100]
	Dietary estimated	1,100,910 participants 1093 developed ALS	β-carotene and lutein inversely associated with ALS risk	Fitzgerald et al. [86]
	Dietary estimated	1329 PD patients	ß-carotene associated with a lower risk of PD	Yang et al. [84]
	Dietary estimated	249 PD patients 348 controls	Higher consumption β-carotene associated with reduced risk of PD	Miyake et al. [93]

FTD, Frontotemporal dementia; MDD, major depression disorder; HDRS-17, Hamilton Depression Rating Scale; MCI, mild cognitive impairment; BPD, bipolar depression.
